# Development of a mentor training curriculum to support LGBTQIA+ health professionals

**DOI:** 10.1017/cts.2024.18

**Published:** 2024-02-15

**Authors:** Brittany M. Charlton, Jennifer Potter, Alex S. Keuroghlian, John L. Dalrymple, Sabra L. Katz-Wise, Carly E. Guss, William R. Phillips, Emeline Jarvie, Shail Maingi, Carl Streed, Ethan Anglemyer, Tabor Hoatson, Bruce Birren

**Affiliations:** 1 Harvard Medical School, Harvard Pilgrim Health Care Institute, Harvard T.H. Chan School of Public Health, Fenway Health, Boston Children’s Hospital, and Brigham and Women’s Hospital, Boston, MA, USA; 2 Harvard Medical School, Beth Israel Deaconess Medical Center, and Fenway Health, Boston, MA, USA; 3 Harvard Medical School, Massachusetts General Hospital, and Fenway Health, Boston, MA, USA; 4 Harvard Medical School and Beth Israel Deaconess Medical Center, Boston, MA, USA; 5 Harvard Medical School, Boston Children’s Hospital, and Harvard T.H. Chan School of Public Health, Boston, MA, USA; 6 Harvard Medical School and Boston Children’s Hospital, Boston, MA, USA; 7 Dana-Farber Cancer Institute, Boston, MA, USA; 8 Boston University School of Medicine, Boston, MA, USA; 9 Harvard Pilgrim Health Care Institute, Boston, MA, USA; 10 Broad Institute of MIT and Harvard University, Cambridge, MA, USA

**Keywords:** Cultural diversity, mentoring, mentors, students, sexual and gender minorities

## Abstract

While mentors can learn general strategies for effective mentoring, existing mentorship curricula do not comprehensively address how to support marginalized mentees, including LGBTQIA+ mentees. After identifying best mentoring practices and existing evidence-based curricula, we adapted these to create the Harvard Sexual and Gender Minority Health Mentoring Program. The primary goal was to address the needs of underrepresented health professionals in two overlapping groups: (1) LGBTQIA+ mentees and (2) any mentees focused on LGBTQIA+ health. An inaugural cohort (*N* = 12) of early-, mid-, and late-career faculty piloted this curriculum in spring 2022 during six 90-minute sessions. We evaluated the program using confidential surveys after each session and at the program’s conclusion as well as with focus groups. Faculty were highly satisfied with the program and reported skill gains and behavioral changes. Our findings suggest this novel curriculum can effectively prepare mentors to support mentees with identities different from their own; the whole curriculum, or parts, could be integrated into other trainings to enhance inclusive mentoring. Our adaptations are also a model for how mentorship curricula can be tailored to a particular focus (i.e., LGBTQIA+ health). Ideally, such mentor trainings can help create more inclusive environments throughout academic medicine.

## Introduction

There is a national focus on improving mentorship and optimizing mentoring relationships. The National Academies of Sciences, Engineering, and Medicine recently released a consensus study on evidence-based approaches to mentorship in STEMM (science, technology, engineering, mathematics, and medicine) [1]. This study, and other published evidence, reveals that quality mentorship leads to improved outcomes across disciplines and career stages, including a sense of belonging, self-efficacy, persistence, productivity, career satisfaction, and academic success, and highlights the relevance of social identities in mentorship. Funding agencies are increasingly calling for, and mandating, evidence-based mentor training [1].

Little is known about mentorship for lesbian, gay, bisexual, transgender, queer, intersex, asexual, and all sexual and gender minority (LGBTQIA+) trainees. This includes trainees working on various topics within clinical care, education, and research. Many of these trainees are multiply marginalized based on their gender identity, sexual orientation, race/ethnicity, and other axes of social inequality. Additionally, many LGBTQIA+ trainees along with their allies choose to focus their work on LGBTQIA+ populations. There is robust evidence of mentorship disparities based on gender and race/ethnicity; presumably, LGBTQIA+ trainees and their allies focused on LGBTQIA+ health are exponentially burdened by these mentorship inequities. Identities matter in mentorship, particularly for mentees historically underrepresented or excluded from medicine. Faculty rate male job applicants as more competent and deserving of mentorship; male applicants are offered ∼$4,000 more in salary and career mentoring than identical female applicants [2]. When male faculty make hiring decisions, they are less likely to hire and train women [3]. Further, mentorship requests from women and racial/ethnic minorities are more often ignored than requests from White men; as a result, these trainees receive less mentoring than their White male peers [4,5]. Such mentorship inequities pose significant obstacles to career development. For example, White investigators are more likely than racial/ethnic minority investigators to win National Institutes of Health R01 awards; inadequate mentoring poses a significant obstacle to obtaining that funding [6].

Even experienced mentors learn strategies for more effective mentoring from existing curricula, such as those from the National Research Mentoring Network and the Center for the Improvement of Mentored Experiences in Research (CIMER) [7]. Research indicates that compared to untrained mentors, those who participate in these curricula observe marked improvements in their mentees’ skills and communication [8]. Moreover, mentees indicate they have a better experience with trained mentors than with untrained mentors [8]. Existing curricula address some ways mentorship can help underrepresented trainees overcome barriers [9]. However, these curricula do not comprehensively address the needs of LGBTQIA+ individuals, particularly those who are multiply marginalized (e.g., transgender women of color). Mentors must help these trainees navigate unique issues like decision-making around disclosing one’s sexual orientation or gender identity in a job interview. Regardless of one’s identity, existing curricula do not address the unique obstacles trainees face who focus on LGBTQIA+ populations in their clinical care, education, or research (e.g., how to assess a prospective employer’s climate). Even for mentors who may not have mentees who are LGBTQIA+ or focused on this population, such training is necessary so faculty can help any marginalized trainee overcome the systemic disadvantage and discrimination that persist throughout academic medicine.

To address some of the needs of LGBTQIA+ health professionals, our team adapted various best mentoring practices and evidence-based curricula (e.g., *Entering Mentoring* ^8^) into the Harvard Sexual and Gender Minority Health Mentoring Program. We hypothesized that faculty participants in our formal training program would observe marked improvements in their mentoring skills by the conclusion of the training. Herein, we describe the process of creating and piloting this curriculum and highlight challenges and solutions to inform similar programs.

## Methods

We conducted interviews with 36 experts across the country; these included organizational leaders, mentorship scholars, and LGBTQIA+ health experts. Experts were identified in part by the Harvard’s LGBTQIA+ medical education initiative’s Professional Advisory Council [10]. The primary goal of these interviews was to identify mentorship needs of LGBTQIA+ health professionals. After conducting these interviews, our mentorship program founder, Dr Brittany Charlton, completed a week-long training to become a CIMER Trained Facilitator; she then led several trainings and became a CIMER Certified Facilitator. Finally, she designed our program’s curriculum and solicited iterative stakeholder feedback.

The inaugural cohort of faculty mentors piloted our curriculum in spring 2022 through a series of 90-minute sessions delivered over six consecutive weeks. The curriculum leverages a case-based approach and helps faculty explore a framework for mentoring, develop new mentoring skills, and create a forum to solve mentoring dilemmas and share strategies for success. Other opportunities are built into the program for peer mentorship, including forming traditional dyads and other mentor structures (e.g., peer mentor groups). Like *Entering Mentoring*, the curriculum is structured around core competencies: (1) aligning mentor–mentee expectations, (2) maintaining effective communication, (3) addressing equity and inclusion, (4) assessing mentee understanding, (5) promoting professional development, (6) fostering independence, and (7) developing a reflective approach to mentoring. Tools include mentoring agreements, individual development plans, and mentor maps. Unlike *Entering Mentoring*, we designed the curriculum not just for researchers but also for those focused on clinical care and education (e.g., medical education).

While the competencies and tools are helpful for any mentor, we tailored the curriculum to meet the needs of two distinct, but often overlapping groups: (1) LGBTQIA+ mentees and (2) any mentee focused on LGBTQIA+ health. We provided an individualized development plan (see Supplemental Exhibits) that mentors could use with mentees; this includes a needs assessment where mentees focused on LGBTQIA+ health rate their proficiency in areas that may be relevant to their work. These tools demonstrate how individualized development plans can be tailored to meet the needs of a particular group, and these tools can also be immediately leveraged by LGBTQIA+ health professionals and their mentors. Relevant case studies were adapted from existing *Entering Mentoring* cases (Table 1), while others were newly developed (Table 2). All *Entering Mentoring* resources are freely available online from CIMER, which may also house our program’s adapted cases in the future. Beyond synchronous learning sessions, faculty committed ∼60 minutes/week to asynchronous activities (e.g., developing mentoring agreements). We utilized Canvas as our Learning Management System, which enabled participants to submit asynchronous activities for peer and facilitator feedback.

**Table 1.** Example of *entering mentoring* case study tailored for LGBTQIA + health professionals

Existing *Entering Mentoring* case study titled “Is it Okay to Ask?”
Last year I worked with a fantastic scholar who has since left to work at another institution. I think that she had a positive experience working with our research team, but a few questions still linger in my mind. This particular scholar was a young African-American woman. I wondered how she felt about being the only African-American woman in our research group. In fact, she was the only African-American woman in our entire department. I wanted to ask her how she felt, but I worried it might be insensitive or politically incorrect to do so. I never asked. I still wonder how she felt and how those feelings may have affected her experience, but I could never figure out how to broach the subject.
**Tailored case study for LGBTQIA+ health professionals**
Dr Caniglia (he/him) is the head of a large research team focused on the use of Pre-Exposure Prophylaxis (PrEP) to prevent HIV. Last year, he worked with a fantastic scholar who has since left to work at another institution. He thinks that she had a positive experience working with the research team, but a few questions still linger in his mind. This scholar was a young, Black transgender woman. He wondered how she felt about being the only person of color, not to mention the only transgender person, on the research team. In fact, she was the only Black transgender woman in the entire department. He wanted to ask her how she felt, but he worried it might be insensitive or inappropriate to do so. He never asked. He still wonders how she felt and how those feelings may have affected her experience, but he could never figure out how to broach the subject.

**Table 2.** Examples of new case studies that address unique challenges for LGBTQIA+-identified trainees or their allies focused on LGBTQIA+ health

New case study titled “Coming Out”
Palmsten (he/him) is a gay, fourth-year medical student who is preparing for residency program interviews. He wants to pursue dermatology and hopes to include LGBTQIA+ patients in his practice and focus his research on related health disparities.
During his medical school interviews, he spoke about his interest in serving LGBTQIA+ communities. However, some faculty interviewers made stigmatizing remarks. Palmsten is unsure about how to handle this situation in his upcoming residency interviews. He is unsure about discussing these interests and doesn't know if he should come out about his own sexual orientation. His mentor is a late-career, lesbian woman so Palmsten asks her how she handled this issue in her own career.
The mentor offers some ideas about how Palmsten can describe motivating factors that brought him to this work and ways he might assess the institutional climate. However, she doesn't know how to best advise him. She’s also struggling to share her own experience as she did not come out until mid-career and feels both guilt about that decision as well as pain in subsequently facing discrimination and stigma.
**New case study titled “To Stay or To Go”**
Dr Feliciano (they/them) has been an instructor in the Division of Cardiovascular Medicine for 7 years. Their job includes 7 clinical sessions, 2 sessions precepting residents, and 1 administrative session. They are known for their skill in teaching about sexual and gender minority health; they are frequently tapped to speak at their own institution as well as at other institutions around the region. The residents and fellows have also chosen them four times for the annual teaching award.
At Dr Feliciano’s annual performance review, they are told that the only promotion they’ll be eligible for at 10 years is a one-step move from instructor to assistant professor according the “longer service criteria.” They feel undervalued and unsupported by the institution and are looking at other jobs.

We used a mixed-methods evaluation approach throughout the program, including anonymous feedback on what worked well or could be changed in a particular session. At the end of the program, also we administered a REDCap survey assessing demographics, workshop satisfaction, behavior changes, and mentoring skill gains using validated assessments, including the Mentoring Competency Assessment (MCA) [11]. In alignment with prior research, we assessed the 26-item MCA at the training’s conclusion. Participants rated their skills at the onset of training (i.e., retrospective pretest) and then at present (i.e., posttest) for each scale item, which aligned with the curriculum’s core competencies. We tested for changes in retrospective pretest to posttest scores using Wilcoxon rank-sum tests. To triangulate these quantitative data, participants also attended one of two focus groups lasting 90 minutes; a semi-structured interview guide included open-ended questions and detailed probes. Audio from the focus groups was professionally transcribed. In line with a template approach [12], two coders independently reviewed the transcripts to identify emergent concepts. This study was approved by the Harvard Pilgrim Health Care Institute Institutional Review Board.

## Results

As hypothesized, we found that faculty participants in our formal training program observed marked improvements in their mentoring skills by the conclusion of the training. Faculty were highly satisfied with the program and reported skill gains and behavioral changes. Among the 12 faculty participants, two-thirds identified as cisgender women (*n* = 8) and one-third as cisgender men (*n* = 4); none identified as another gender (e.g., gender fluid, nonbinary). The majority identified their sexual orientation as gay/lesbian (58%, *n* = 7), while others identified as heterosexual (*n* = 2), bisexual (*n* = 1), pansexual (*n* = 1), and queer (*n* = 1). One participant was Black, another was Asian Indian, and the rest were non-Hispanic White. Regarding academic rank, one-third were instructors, another third were assistant professors, one-quarter were associate professors, and one was a full professor. Nine participants held MD degrees, while three had PhD or equivalent research degrees. Mentoring experience varied; some participants had 21+ years of mentoring (33%, *n* = 4), and one had no experience. Roughly half had completed prior mentor training.

Ten of the twelve participants (83%) completed all evaluations, including reports of workshop satisfaction and mentoring skill gains. All participants reported the training was a valuable use of time, and 100% said they were “very likely” or “likely” to recommend the training to a colleague. When asked about changes to their mentoring, 100% noted they had already implemented changes based on the training.

Qualitative data supported the quantitative findings; for example, participants spoke about changing how they communicated with their mentees and using individual development plans and mentoring agreements. When describing such changes, one participant commented, “I will be much more explicit, from the beginning, with my mentees. I will use a mentor agreement to detail expectations. I am more aware of power differentials and how those may put mentees in difficult positions, even if well-intentioned by the mentor and I am more thoughtful about how requests and conversations may be experienced by mentees, especially those from marginalized groups.” Another noted, “I plan to articulate for myself more clearly the role I play in various mentoring relationships. I will try to communicate those expectations clearly to my mentees and make clear any expectations I have for them. I will use the tools from this program to think about how I can foster mentee career development and how I can foster growth and independence.”

**Figure 1.**
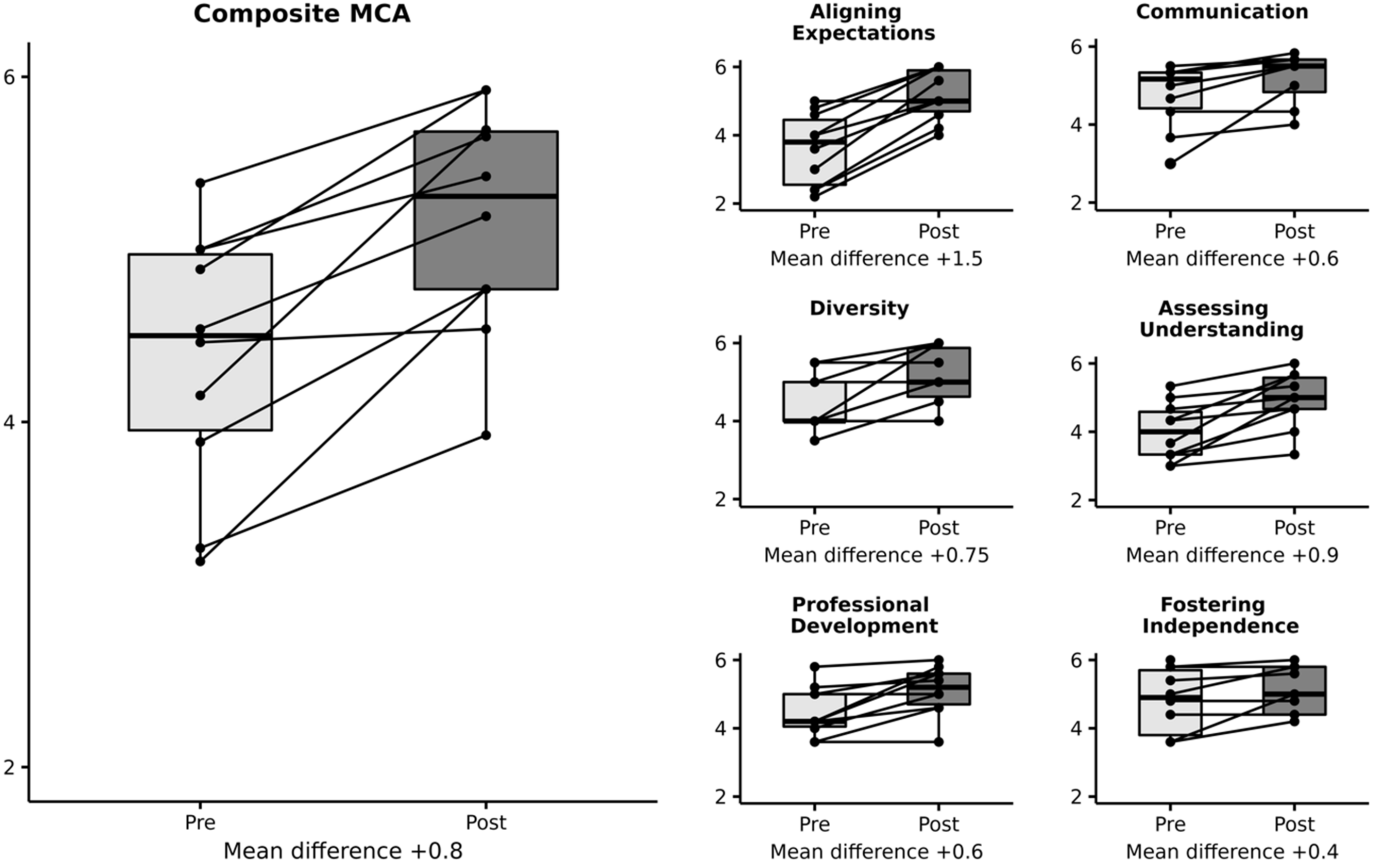
Comparison of mentoring competency assessment (MCA)^1^ scores before and after participants completed the Harvard Sexual and Gender Minority Health Mentoring Program. ^1^MCA scores were assessed at the training’s conclusion; all differences are statistically significant (*p* < 0.02). Participants rated their skills at the onset of training (i.e., retrospective pretest) and then at present (i.e., posttest) for 26 items, each of which was aligned with one of the curriculum’s six core competencies: (1) aligning mentor–mentee expectations, (2) maintaining effective communication, (3) addressing equity and inclusion, (4) assessing mentee understanding, (5) promoting professional development, and (6) fostering independence.

Participants rated their mentoring skill levels on a seven-point Likert scale (1 = not at all skilled, 4 = moderately skilled, 7 = extremely skilled). Participants reported significant gains in the quality of their mentoring (4.1–5.2; +1.1, *p* < 0.01), confidence in their mentoring ability (4.4–5.4; +1.1, *p* < 0.01), and their ability to meet mentee expectations (4.2–5.1; +0.9; *p* < 0.01). The mean change in MCA composite scores from the retrospective pretest to posttest was + 0.8 (4.38–5.18, *p* < 0.01, see Fig. 1). As an assessment of the core competencies, all six subscale scores significantly improved (communication + 0.6; expectations + 1.5; understanding + 0.9; independence + 0.4; diversity + 0.75; and professional development + 0.6; *p* < 0.02).

## Discussion

The status quo for mentor training in academic medicine is an *ad hoc* approach [1]. The Harvard Sexual and Gender Minority Health Mentoring Program is a substantive, innovative departure due to its structured, longitudinal, evidence-based approach. To our knowledge, this is the first mentor training focused on LGBTQIA+ trainees. Our pilot data suggest this curriculum could be an effective way to help any mentor in a position to support mentees with different identities from their own, this includes LGBTQIA+ trainees and beyond. The whole curriculum, or parts, could be integrated into other trainings to enhance inclusive mentoring; for example, trainers could use the *Entering Mentoring* adapted case titled “Is It Ok To Ask?” to discuss unique issues that impact transgender women of color working on a topic that heavily burdens their community (i.e., HIV, see Table 1). Our adaptations are also a model for how curriculum can be tailored for a particular topic area (i.e., LGBTQIA+ health).

We believe that disseminating this curriculum can help to improve the broader medical field. As a precursor to our mentor training pilot, part of our team led the creation of three parallel mentor programs through the Harvard Sexual Orientation and Gender Identity and Expression (SOGIE) Health Equity Research Collaborative, which is a hub for LGBTQIA+ health research at the university and its teaching hospitals. While these programs do not include formal mentor training, they provide peer mentorship for faculty, postdoctoral fellows, and graduate students; this curriculum can complement these programs. This curriculum will also be offered as part of our newly launched LGBTQIA+ Health Fellowship Program sponsored by the American Medical Association Foundation. Several participants in our inaugural mentoring cohort lead existing faculty development programs across Harvard, where our curricula also can be integrated. Beyond Harvard, we have shared this curriculum with professional societies, such as at the GLMA Annual Conference on LGBTQ Health, the Society for Epidemiologic Research, and the American Association for the Advancement of Science. This curriculum can fill a unique gap as the broader health disparity field grows, including with the addition of training grants, some of which are focused on LGBTQIA+ health.

The program requires resources including human capital. Sessions should be led by one or two facilitators trained through an organization such as CIMER. Facilitators should also have experience working on diversity, equity, and inclusion topics like LGBTQIA+ health. Compensation is also necessary for facilitators and staff to prepare, deliver, and evaluate trainings. Ideally, such a program would be housed within an existing entity, such as a Mentoring Center or Core, to ensure it is scalable and sustainable.

Dr Charlton was recently granted one of the inaugural awards from the National Institutes of Health for excellence in diversity, equity, and inclusion-focused mentorship. One aim of that award is to expand this program to develop, evaluate, refine, and disseminate two additional mentor training curricula, one for residents/fellows/postdoctoral fellows and another for medical/graduate students. The faculty curriculum already has activities in traditional dyads and peer groups. With the addition of two cohorts, we can incorporate even more collective mentoring structures across ranks. We can also scale up our programing, such as hosting regular seminars and networking events across the three cohorts and among alums.

The program and evaluation are not without limitations. While the size of the inaugural faculty cohort was chosen to optimize the experience of the participants and facilitator, that size limits the generalizability. Subsequent evaluations with many more cohorts in other institutions across the country can, for example, elucidate how the program and evaluations differ based on whether the participant is a member of the LGBTQIA+ community. Larger samples can also examine variation among participants with prior mentor training experience or those with a lengthy track record of mentorship. Particularly because our program founder facilitated the program and conducted the evaluations, participants may have reported more positive outcomes due to social desirability bias; as more mentor training facilitators use this curriculum, we can more formally assess for this bias. In line with the Kirkpatrick model [13], subsequent research could assess long-term outcomes from mentors who complete this curriculum as well as their mentees.

Mentor training curricula must recognize and respond to diverse identities. Without such curricula, along with additional institutional commitments to diversity, equity, and inclusion, underrepresented individuals will continue to be burdened by disadvantage and discrimination, negatively impacting their careers and the entire scientific and medical enterprise. While this was a formative process evaluation, the long-term goal is that each mentor who completes this curriculum will improve their dyadic mentoring relationship and influence the mentoring environment around them, ultimately improving the systems within which these exist. It is our hope that this curriculum can help to positively impact the quality of mentorship among LGBTQIA+ health professionals and therefore increase the quality of clinical care, education, and research with this population; these innovations are particularly needed in the burgeoning LGBTQIA+ health field and throughout academic medicine.

## Supporting information

Charlton et al. supplementary material Charlton et al. supplementary material

## Supplementary material

The supplementary material for this article can be found at https://doi.org/10.1017/cts.2024.18.
