# Supplementary material for: Development of a mentor training curriculum to support LGBTQIA+ health professionals
Source: J Clin Transl Sci. 2024 Feb 15;8(1):e44. doi: 10.1017/cts.2024.18 (PMC10928698; doi:10.1017/cts.2024.18)
Supplement: Charlton et al. supplementary material [file S2059866124000189sup001.docx]

**HARVARD SEXUAL AND GENDER MINORITY MENTORING PROGRAM**

**SELF-ASSESSMENT AND INDIVIDUAL DEVELOPMENT PLAN**

**STEP 1: SELF-ASSESSMENT**

This self-assessment is designed to help you think about your skills, values, strengths, and challenges. You will share this self-assessment with your mentor to get feedback and request assistance in your development. Additionally, this self-assessment will guide your individual development plan (IDP), which you will draft in Step 2.

**Step 1A—Skills**

For each skill, you will rate your proficiency and commitment to developing in the next year. These skills can be used throughout various career stages and across disciplines/areas of expertise/specialties, so if a skill does not apply, you can indicate it is not applicable (N/A).

|  | ***Rate your proficiency***  ***1=Needs improvement***  ***5=Highly Proficient*** | | | | | |  | ***Would you commit time to develop this skill in the next year?*** | |
| --- | --- | --- | --- | --- | --- | --- | --- | --- | --- |
| **Clinical** |  |  |  |  |  |  |  |  |  |
| Skill/knowledge area:__________________________ | N/A | 1 | 2 | 3 | 4 | 5 |  | Y | N |
| Skill/knowledge area:__________________________ | N/A | 1 | 2 | 3 | 4 | 5 |  | Y | N |
| Case management | N/A | 1 | 2 | 3 | 4 | 5 |  | Y | N |
| Efficiency | N/A | 1 | 2 | 3 | 4 | 5 |  | Y | N |
| Other: | N/A | 1 | 2 | 3 | 4 | 5 |  | Y | N |
|  |  |  |  |  |  |  |  |  |  |
| **Research** |  |  |  |  |  |  |  |  |  |
| Turn your work into academic scholarship | N/A | 1 | 2 | 3 | 4 | 5 |  | Y | N |
| Perform literature review | N/A | 1 | 2 | 3 | 4 | 5 |  | Y | N |
| Construct hypothesis and aims | N/A | 1 | 2 | 3 | 4 | 5 |  | Y | N |
| Develop novel research questions | N/A | 1 | 2 | 3 | 4 | 5 |  | Y | N |
| Understand study designs | N/A | 1 | 2 | 3 | 4 | 5 |  | Y | N |
| Design a study design to meet the hypothesis and aims | N/A | 1 | 2 | 3 | 4 | 5 |  | Y | N |
| Navigate co-authorship | N/A | 1 | 2 | 3 | 4 | 5 |  | Y | N |
| IRB submission and process | N/A | 1 | 2 | 3 | 4 | 5 |  | Y | N |
| Research computing skills (e.g., citation, analytic software) | N/A | 1 | 2 | 3 | 4 | 5 |  | Y | N |
| Quantitative research skills | N/A | 1 | 2 | 3 | 4 | 5 |  | Y | N |
| Understand statistical analysis | N/A | 1 | 2 | 3 | 4 | 5 |  | Y | N |
| Prepare a quantitative analysis proposal | N/A | 1 | 2 | 3 | 4 | 5 |  | Y | N |
| Conduct statistical analysis | N/A | 1 | 2 | 3 | 4 | 5 |  | Y | N |
| Qualitative research skills | N/A | 1 | 2 | 3 | 4 | 5 |  | Y | N |
| Prepare a qualitative analysis proposal | N/A | 1 | 2 | 3 | 4 | 5 |  | Y | N |
| Conduct a qualitative analysis | N/A | 1 | 2 | 3 | 4 | 5 |  | Y | N |
| Engage stakeholders (e.g., community-based participatory research) | N/A | 1 | 2 | 3 | 4 | 5 |  | Y | N |
| Prepare a review article or expert opinion | N/A | 1 | 2 | 3 | 4 | 5 |  | Y | N |
| Prepare a manuscript | N/A | 1 | 2 | 3 | 4 | 5 |  | Y | N |
| Disseminate work (e.g., identify presentation, publication outlets) | N/A | 1 | 2 | 3 | 4 | 5 |  | Y | N |
| Respond to reviewers’ critiques and revise a manuscript | N/A | 1 | 2 | 3 | 4 | 5 |  | Y | N |
| Review and critique manuscripts for a journal | N/A | 1 | 2 | 3 | 4 | 5 |  | Y | N |
| Creatively developing new research directions | N/A | 1 | 2 | 3 | 4 | 5 |  | Y | N |
| Find funding opportunities | N/A | 1 | 2 | 3 | 4 | 5 |  | Y | N |
| Write grant proposals | N/A | 1 | 2 | 3 | 4 | 5 |  | Y | N |
| Other: | N/A | 1 | 2 | 3 | 4 | 5 |  | Y | N |
|  |  |  |  |  |  |  |  |  |  |
| **Teaching** |  |  |  |  |  |  |  |  |  |
| Create/design oral presentations for research (e.g., conferences) | N/A | 1 | 2 | 3 | 4 | 5 |  | Y | N |
| Create/design oral presentations for teaching | N/A | 1 | 2 | 3 | 4 | 5 |  | Y | N |
| Deliver oral presentations | N/A | 1 | 2 | 3 | 4 | 5 |  | Y | N |
| Teach one-on-one | N/A | 1 | 2 | 3 | 4 | 5 |  | Y | N |
| Teach in small groups | N/A | 1 | 2 | 3 | 4 | 5 |  | Y | N |
| Teach in large groups | N/A | 1 | 2 | 3 | 4 | 5 |  | Y | N |
| Utilize evidenced-based teaching skills | N/A | 1 | 2 | 3 | 4 | 5 |  | Y | N |
| Provide feedback to learners | N/A | 1 | 2 | 3 | 4 | 5 |  | Y | N |
| Develop a syllabus/curriculum | N/A | 1 | 2 | 3 | 4 | 5 |  | Y | N |
| Develop a lesson plan | N/A | 1 | 2 | 3 | 4 | 5 |  | Y | N |
| Obtain peer review of your teaching | N/A | 1 | 2 | 3 | 4 | 5 |  | Y | N |
| Other: | N/A | 1 | 2 | 3 | 4 | 5 |  | Y | N |
|  |  |  |  |  |  |  |  |  |  |

|  | ***Rate your proficiency***  ***1=Needs improvement***  ***5=Highly Proficient*** | | | | | |  | ***Would you commit time to develop this skill in the next year?*** | |
| --- | --- | --- | --- | --- | --- | --- | --- | --- | --- |
| **Mentoring** |  |  |  |  |  |  |  |  |  |
| Identify, approach, and utilize mentors | N/A | 1 | 2 | 3 | 4 | 5 |  | Y | N |
| Negotiate and maintain a mentoring relationship | N/A | 1 | 2 | 3 | 4 | 5 |  | Y | N |
| Set and achieve goals and timelines | N/A | 1 | 2 | 3 | 4 | 5 |  | Y | N |
| Mentor junior colleagues or trainees | N/A | 1 | 2 | 3 | 4 | 5 |  | Y | N |
| Other: | N/A | 1 | 2 | 3 | 4 | 5 |  | Y | N |
|  |  |  |  |  |  |  |  |  |  |
| **Career Development** |  |  |  |  |  |  |  |  |  |
| Navigate organizational/institutional culture | N/A | 1 | 2 | 3 | 4 | 5 |  | Y | N |
| Prepare your CV and keep it up to date | N/A | 1 | 2 | 3 | 4 | 5 |  | Y | N |
| Join professional societies | N/A | 1 | 2 | 3 | 4 | 5 |  | Y | N |
| Identify and obtain leadership positions (e.g., professional societies, editorial boards) | N/A | 1 | 2 | 3 | 4 | 5 |  | Y | N |
| Enhance your visibility (at the local, regional, national, and international levels) | N/A | 1 | 2 | 3 | 4 | 5 |  | Y | N |
| Understand promotion criteria | N/A | 1 | 2 | 3 | 4 | 5 |  | Y | N |
| Set a direction to complete promotion requirements | N/A | 1 | 2 | 3 | 4 | 5 |  | Y | N |
| Prepare your dossier | N/A | 1 | 2 | 3 | 4 | 5 |  | Y | N |
| Balance personal and professional life | N/A | 1 | 2 | 3 | 4 | 5 |  | Y | N |
| Other: | N/A | 1 | 2 | 3 | 4 | 5 |  | Y | N |
|  |  |  |  |  |  |  |  |  |  |
| **Leadership, Management, and Interpersonal** |  |  |  |  |  |  |  |  |  |
| Time management (e.g., workload, planning, pace of career) | N/A | 1 | 2 | 3 | 4 | 5 |  | Y | N |
| Collegiality | N/A | 1 | 2 | 3 | 4 | 5 |  | Y | N |
| Written communication | N/A | 1 | 2 | 3 | 4 | 5 |  | Y | N |
| Verbal communication | N/A | 1 | 2 | 3 | 4 | 5 |  | Y | N |
| Awareness of communication styles (yours and others') | N/A | 1 | 2 | 3 | 4 | 5 |  | Y | N |
| Networking (e.g., creating and maintaining professional networks) | N/A | 1 | 2 | 3 | 4 | 5 |  | Y | N |
| Negotiation (e.g., to achieve career goals) | N/A | 1 | 2 | 3 | 4 | 5 |  | Y | N |
| Conflict resolution | N/A | 1 | 2 | 3 | 4 | 5 |  | Y | N |
| Lead and motivate others | N/A | 1 | 2 | 3 | 4 | 5 |  | Y | N |
| Lead diverse teams | N/A | 1 | 2 | 3 | 4 | 5 |  | Y | N |
| Chair a committee, task force, or small group | N/A | 1 | 2 | 3 | 4 | 5 |  | Y | N |
| Manage projects and programs | N/A | 1 | 2 | 3 | 4 | 5 |  | Y | N |
| Delegate responsibility | N/A | 1 | 2 | 3 | 4 | 5 |  | Y | N |
| Create and manage budgets | N/A | 1 | 2 | 3 | 4 | 5 |  | Y | N |
| Receive and use feedback from others | N/A | 1 | 2 | 3 | 4 | 5 |  | Y | N |
| Provide feedback to others | N/A | 1 | 2 | 3 | 4 | 5 |  | Y | N |
| Other: | N/A | 1 | 2 | 3 | 4 | 5 |  | Y | N |

|  | ***Rate your proficiency***  ***1=Needs improvement***  ***5=Highly Proficient*** | | | | | |  | ***Would you commit time to develop this skill in the next year?*** | |
| --- | --- | --- | --- | --- | --- | --- | --- | --- | --- |
| **Sexual and Gender Minority Health** |  |  |  |  |  |  |  |  |  |
| Define and describe the **differences** among: sex and gender; gender identity and gender expression; gender diversity and gender dysphoria; and sexual orientation-related attraction, behavior, and identity | N/A | 1 | 2 | 3 | 4 | 5 |  | Y | N |
| Define and describe specific **health inequities** related to sexual orientation, gender identity and expression, and sex development, and how these intersect with health experiences related to race, ethnicity, gender, age, ability, education, economic status, immigration status, and other social positions. | N/A | 1 | 2 | 3 | 4 | 5 |  | Y | N |
| Outline strategies that can address sexual and gender minority health inequities at the individual, organizational, community, and societal levels based on the **social-ecological model.** | N/A | 1 | 2 | 3 | 4 | 5 |  | Y | N |
| Develop **rapport** with all patients, families, and/or members of the health care team regardless of their gender identities, gender expressions, sexual orientations, and sex development, to promote respectful and affirming interpersonal exchanges, including staying current with evolving terminology and practice guidelines and recommendations. | N/A | 1 | 2 | 3 | 4 | 5 |  | Y | N |
| Recognize how **unconscious bias** and assumptions about sexual and gender minority people may negatively affect clinical encounters and healthcare outcomes and develop strategies to mitigate these effects. | N/A | 1 | 2 | 3 | 4 | 5 |  | Y | N |
| Recognize how inherent **power imbalances** between physician and patient or between physician and parent/guardian, including dynamics related to race, ethnicity, gender, age, ability, education, economic status, immigration status, and other social positions may negatively affect clinical encounters and outcomes for sexual and gender minority patients and develop strategies to mitigate these imbalances. | N/A | 1 | 2 | 3 | 4 | 5 |  | Y | N |
| Develop **rapport** with all patients, families, and/or members of the health care team regardless of their gender identities, gender expressions, sexual orientations, and sex development, to promote respectful and affirming interpersonal exchanges, including staying current with evolving terminology and practice guidelines and recommendations. | N/A | 1 | 2 | 3 | 4 | 5 |  | Y | N |
| Sensitively and effectively **elicit relevant information** about sex anatomy, sex development, sexual behavior, sexual history, sexual orientation, and gender identity from all patients in a confidential and developmentally appropriate manner. | N/A | 1 | 2 | 3 | 4 | 5 |  | Y | N |
| Perform a complete and accurate **physical exam with sensitivity** to issues specific to sexual and gender minority patients at all stages across the lifespan. This includes knowing when components of the exam are essential and when they may be unnecessarily traumatizing or require adaptation. | N/A | 1 | 2 | 3 | 4 | 5 |  | Y | N |
| Demonstrate the ability to **individualize screening** and tailor trauma-informed clinical care according to a patient’s preferences, identities, anatomical structures present, and biobehavioral risk factors. | N/A | 1 | 2 | 3 | 4 | 5 |  | Y | N |

**Step 1B—Values**

Values are the principles by which we live. Your values can help define the environment in which you are likely to function best, and the conditions of a job that will keep you motivated.

For each value, rate how important it is to you that your career path matches each of the following values. Do not rate all of the values as “essential,” try to be discriminating and think about which values genuinely are the *most important* to you

| **Value** | **Description** | ***Rate importance***  ***1=Unimportant***  ***5=Essential*** | | | | | |
| --- | --- | --- | --- | --- | --- | --- | --- |
| Help society | Do something to contribute to the betterment of the world. | N/A | 1 | 2 | 3 | 4 | 5 |
| Help others | Be involved in directly helping other people either individually or in small groups. | N/A | 1 | 2 | 3 | 4 | 5 |
| People contact | Have a lot of day-to-day contact with people due to my work activities. | N/A | 1 | 2 | 3 | 4 | 5 |
| Teamwork | Work in collaboration with others or as part of a team. | N/A | 1 | 2 | 3 | 4 | 5 |
| Affiliation | Be recognized as a member of a respected organization. | N/A | 1 | 2 | 3 | 4 | 5 |
| Friendships | Develop close personal relationships with people due to my work activities; or have a job that allows time to maintain close friendships outside of work. | N/A | 1 | 2 | 3 | 4 | 5 |
| Competition | Engage in activities that pit (or rank) my abilities or achievements against the abilities or achievements of others. | N/A | 1 | 2 | 3 | 4 | 5 |
| Make decisions | Have the power to decide courses of action, policies, etc. | N/A | 1 | 2 | 3 | 4 | 5 |
| Work under pressure | Work in situations where time pressure is prevalent and/or the quality of my work is judged critically by supervisors, customers, or others. | N/A | 1 | 2 | 3 | 4 | 5 |
| Power and authority | Control the work activities of other people. | N/A | 1 | 2 | 3 | 4 | 5 |
| Influence people | Be in a position to change the attitudes or opinions of other people. | N/A | 1 | 2 | 3 | 4 | 5 |
| Work alone | Work on or complete projects alone, without any significant contact with others. | N/A | 1 | 2 | 3 | 4 | 5 |
| Knowledge | Engage me in pursuing knowledge, truth, and understanding; intellectual stimulation. | N/A | 1 | 2 | 3 | 4 | 5 |
| Intellectual or expert status | Be regarded as a person of high intellectual prowess or as an acknowledged expert in a given field. | N/A | 1 | 2 | 3 | 4 | 5 |
| Creativity | Create new ideas, programs, organizational structures, or anything else not following a format previously developed by others. | N/A | 1 | 2 | 3 | 4 | 5 |
| Aesthetics | Be involved in studying, appreciating the beauty of things, ideas, etc., or creating that beauty. | N/A | 1 | 2 | 3 | 4 | 5 |
| Supervision | Having a job in which I am directly responsible for the work done by others. | N/A | 1 | 2 | 3 | 4 | 5 |
| Stability | Have a work routine and job duties that are largely predictable and not likely to change over a long period. | N/A | 1 | 2 | 3 | 4 | 5 |
| Change and variety | Have work responsibilities that frequently change their content and setting; avoidance of routine. | N/A | 1 | 2 | 3 | 4 | 5 |
| Precision work | Work in a situation where there is very little tolerance for error. | N/A | 1 | 2 | 3 | 4 | 5 |
| Security | Be assured of keeping my job and a reasonable financial reward. | N/A | 1 | 2 | 3 | 4 | 5 |
| Fast pace | Work in settings with a high and/or rapid activity rate. | N/A | 1 | 2 | 3 | 4 | 5 |
| Recognition | Be recognized (by colleagues or patients or the public or superiors) for the quality of my work. | N/A | 1 | 2 | 3 | 4 | 5 |
| Excitement | Experience a high degree of (or frequent) excitement during my work. | N/A | 1 | 2 | 3 | 4 | 5 |
| Adventure | Work duties involving risk-taking or trying new things; could be a position that allows a lot of travel. | N/A | 1 | 2 | 3 | 4 | 5 |
| High earnings anticipated | Monetary rewards will be such that I can purchase those things I consider essential as well as the luxuries of life that I wish to have. | N/A | 1 | 2 | 3 | 4 | 5 |
| Profit-gain | Have a strong likelihood of accumulating a large amount of money or other material gain. | N/A | 1 | 2 | 3 | 4 | 5 |
| Independence | Be able to determine the nature of my work without significant direction from others; not be required to do what others tell me to do. | N/A | 1 | 2 | 3 | 4 | 5 |
| Moral fulfillment | Feel that my work is contributing significantly to, or is in keeping with, a set of moral standards, which I feel are very important. | N/A | 1 | 2 | 3 | 4 | 5 |
| Location | Find a place to live (town, area) that is conducive or accommodating to my lifestyle and allows me to do things I enjoy most. | N/A | 1 | 2 | 3 | 4 | 5 |
| Community involvement | Live in a town or city where I can get involved in community affairs. | N/A | 1 | 2 | 3 | 4 | 5 |
| Physical challenge | Have a job that makes physical demands which I would find rewarding. | N/A | 1 | 2 | 3 | 4 | 5 |
| Time freedom | Have work responsibilities which I can work at according to my time; flexible work schedule or no particular work schedule. | N/A | 1 | 2 | 3 | 4 | 5 |
| Status | The position I have carries respect from my friends, my family, or some community of people. | N/A | 1 | 2 | 3 | 4 | 5 |
| Advancement | A job that provides the opportunity to work hard and make rapid career advancement. | N/A | 1 | 2 | 3 | 4 | 5 |
| Challenging problems | The position does not have to be “essential to the survival of the human race” but should provide challenging problems to solve and avoid continual routine. | N/A | 1 | 2 | 3 | 4 | 5 |
| Creative expression | Opportunity to express in writing or verbally my ideas, reactions, and observations concerning my job and how I might improve it. | N/A | 1 | 2 | 3 | 4 | 5 |
| Job tranquility | To avoid pressure and the “rat race.” | N/A | 1 | 2 | 3 | 4 | 5 |
| Work/life balance | A job that allows me to achieve balance between time spent at work and time spent doing other activities. | N/A | 1 | 2 | 3 | 4 | 5 |
| Family friendly | A job that allows me to balance family and work without threatening my promotion or advancement. | N/A | 1 | 2 | 3 | 4 | 5 |
| Work on the frontiers of knowledge | Do work In an environment that allows me to be at the cutting edge my field. | N/A | 1 | 2 | 3 | 4 | 5 |
| Exercise competence | An opportunity to involve myself in areas where I feel I have talents greater than the average person. | N/A | 1 | 2 | 3 | 4 | 5 |
| Sense of community | Work in an environment that provides a positive sense of professional community | N/A | 1 | 2 | 3 | 4 | 5 |

What additional *rewards or outcomes* do you want to realize from your work that may not be listed above?

Review your rankings. Below, write the values that are ranked as essential.

**Step 1C—Strengths and Challenges**

You will now reflect and describe your strengths and challenges.

Think about areas of strength that you know to be true about yourself. What have others appreciated about you? When did you feel so immersed in a project that you lost track of time and felt success in the activity?

Look hard at those areas that are continually challenging for you. What have others commented that you need to work on?

**STEP 2: INDIVIDUAL DEVELOPMENT PLAN**

You assessed your skills and values in Steps 1A and 1B. Now, you will write an individual development plan (IDP) that helps you to (1) identify long-term career goals, (2) evaluate the past year, and (3) create goals for the next year.

You will follow these iterative and interactive steps to create your IDP:

1. Complete the below IDP template, set it aside for a few days.
2. Review and revise your IDP, ensure goals are SMART
   1. Specific: Have you clarified precisely what needs to happen in each area?
   2. Measurable: Will you know whether you have achieved your objective?
   3. Attainable: Are your goals achievable in light of existing resources (e.g., time, money, skills)?
   4. Result-oriented: Will your IDP help to move you toward your goals?
   5. Time-limited: Does the IDP include realistic timelines?
3. Provide your mentor with a copy of your IDP to review. Expect honest feedback.
4. Reflect on your mentor’s feedback. Where appropriate, integrate your mentor’s suggestions. When choosing not to incorporate the mentor’s suggestions, communicate your decision and why you deem it best to choose another path.
5. Send the revised IDP to your mentor and keep a copy readily accessible as a reminder of your goals and timeline.
6. Implement your IDP and ensure you make necessary revisions throughout the year, as needed.
7. Plan to set an annual (or more frequently, if appropriate) meeting with your mentor to review and discuss your IDP. Prepare a written outline for this discussion; for example, create a prioritized list of the most essential items you wish to discuss.

**Annual Individual Development Plan (IDP)**

**for (year)**

**(Your name)**

Date:

Mentor:

Academic rank:

Years in rank:

Department:

Career Goals
What are your **long-term (3-8 years) career goals**?

What were your **career goals for the last year?** Which of these goals did you meet? If you did not meet a goal, why?

What are you **most proud of** from your work last year?

What are your **career goals for the upcoming year**?

What are the **motivating factors** for pursuing these particular long- and short-term goals?

Are there **special circumstances or barriers** that may make it more challenging to achieve your goals for the upcoming year? What can you do to overcome these challenges?

Network

**Who helped you** in meeting your career goals in the last year?

**Who did you help** in meeting their goals in the last year?

How might your **network** change to help you meet your career goals in the upcoming year?

Time Management

By your best estimate, how did you **allocate** your time during the past year?
% of time spent on clinical care:

% of time spent on research and/or creative work:

% of time spent on teaching, training, or mentoring others:

% of time spent on administration and other duties:

How, if at all, will you **change this time distribution** in the upcoming year?

Institutional Resources

What **institutional resources** have helped you to achieve your goals in the last year?

What **institutional resources** do you need to help you achieve your goals in the upcoming year?

Personal Goals

What are your **personal goals** for the upcoming year?

How will you **accomplish** these personal goals in the upcoming year?

Development of General Research/Scholarly Skills

What additional **research-related skills** do you need to acquire to be successful in this step of your career in the next step?

What will you do during the next year to improve in this area?

What further development do you need in **grant writing, oral presentation of your work, manuscript writing, mentoring, or being a better mentee**?

What will you do during the next year to improve in these areas?

Development of Teaching Skills

What further **teaching skills** do you need to acquire to be successful in this step of your career and the next step?

What will you do during the next year to improve in this area?

Development of Leadership and Management Skills

What further development do you need in **leadership**, budgeting, time management, project management and organization?

What will you do during the next year to improve in these areas?

Development of Interpersonal Skills

What further development do you need in this area of **interpersonal skills**?

What will you do during the next year to improve in this area?

Development of Your CV and Dossier

Update your **CV** and your dossier (if applicable). Attach the updated document(s) to this IDP.

Final Goal Setting and Prioritizing

Overall, what goals will receive your top priority for the coming year?

Create a monthly timeline for fulfilling these goals and attach it to this IDP. Remember to break down each goal for the upcoming year into its smaller, accomplishable sub-goals, steps or “deliverables,” with specific dates for completion. For example:

- - *Major goal: Submit a paper for publication. Completion date - March 2022*
    - *Subgoal #1: Complete data analysis, figures, and outline. Completion date - October 2021*
    - Subgoal *#2: Complete the Introduction section. Completion date - November 2021*
    - *Subgoal #3: Complete the Discussion section. Completion date - December 2021*
